# Study on potential role of apolipoprotein E in recurrent pregnancy loss

**DOI:** 10.3892/etm.2013.997

**Published:** 2013-03-11

**Authors:** ENGIN KORKMAZER, EMIN USTUNYURT, BAŞAR TEKIN, OGUZ CILINGIR

**Affiliations:** 1 Department of Obstetrics and Gynecology, Faculty of Medicine, Eskişehir Osmangazi University, Eskisehir;; 2 Department of Obstetrics and Gynecology, Bursa Şevket Yılmaz Research and Education Hospital, Bursa;; 3 Department of Medical Genetics, Eskişehir Osmangazi University, Faculty of Medicine, Eskisehir, Turkey

**Keywords:** apolipoprotein E, polymorphism, pregnancy, recurrent pregnancy loss

## Abstract

The role of apolipoprotein E (Apo E) gene polymorphisms in the etiology of recurrent pregnancy loss (RPL) is not clearly understood. We evaluated this polymorphism in unexplained pregnancy losses in a group of Turkish women. In our prospective case-control study, 45 well-characterized RPL cases were examined for their Apo E genotypes, based on restriction fragment length polymorphism analysis of polymerase chain reaction (PCR)-amplified fragments. The observed genotypes were compared with those obtained from equal number matched controls. We observed similar Apo E genotypes and E2, E3 and E4 allele frequency distribution among RPL patients and controls. The allele frequencies obtained in patients and controls, respectively, were as follows: E2=8 (9%) and 12 (13.4%) (P=0.342), E3=66 (73.3%) and 60 (66.6%) (P=0.328) and E4=16 (17.7%) and 18 (20%) (P=0.703). Our data did not support the association of Apo E gene polymorphisms with RPL as reported by previous studies. We endorse adequate characterization of RPL cases and adequate sample size prior to addressing such studies.

## Introduction

Recurrent pregnancy losses (RPLs) affect 1% of women and there are a number of factors playing a part. RPL is one of the least understood pathological processes, in spite of being one of the most common reproductive problems. The reason for 68% of RPL cases is idiopathic. In at least 50% of couples with RPL, evaluation, including parental karyotyping, hysterosalpingography or hysteroscopy and antiphospholipid antibody testing may be normal (1).

For the successful progress of pregnancy, an effective uteroplacental circulation is mandatory and this circulation is affected by hemostatic disorders. Considering the fact that hemostatic failures may cause an obstruction of the placental vascular lacunes, unexpected changes in coagulation factors during pregnancy may be the reason for RPL. Pregnancy is a hypercoagulable state characterized by increased levels of prothrombotic factors and decreased antithrombotic factors.

Apolipoprotein E (Apo E) is a protein that plays a key role in the metabolism of cholesterol and triglycerides by binding to receptors in the liver to mediate clearance of chylomicrons and very-low-density lipoproteins from the bloodstream (2). The primary site of synthesis is in the liver; however, other organs and tissues synthesize Apo E, including the brain, spleen, kidneys, gonads, adrenals and macrophages. The Apo E gene has three alleles, ε2 (E2), ε3 (E3) and ε4 (E4), which are on the long (q) arm of chromosome 19 at position 13.2 (3). The Apo E2 allele is associated with decreased serum low-density lipoprotein (LDL) cholesterol, whereas the Apo E4 allele is associated with higher LDL cholesterol. The Apo E4 allele is also thought to be involved in inflammatory responses, platelet function, apoptosis and modulation of oxidative stress (4). The role of Apo E has also been implicated in pregnancy and one study analyzed the effect of Apo E polymorphisms in pre-eclampsia and RPL (5). Therefore, to investigate whether Apo E polymorphisms affect the outcome of pregnancy, we conducted a case-control study. The Apo E allele and genotype distributions of RPL women were compared with those of healthy parous control women.

## Materials and methods

### Patients

A total of 90 women were included in this case-control study. Of these, 45 women had a history of ≥2 consecutive spontaneous abortions with no previous history of successful pregnancy and 45 fertile women had at least one live birth and had no history of spontaneous abortion. Additionally, they had no previous history of pre-eclampsia, preterm delivery, ectopic pregnancy or any other pregnancy-related complications, as well as other non-obstetrical conditions, including chromosomal abnormalities, Mullerian duct defects, abnormal immunological and endocrinological tests and thrombophilia. Women with alcohol usage and smokers were excluded from the study. All the samples were collected from patients attending the outpatient department of the Eskişehir Osmangazi University Department of Gynecology and Obstetrics. All the couples experiencing RPL were evaluated. Chromosome analyses were performed on all subjects. All women with RPL were examined by ultrasonography or hysterosalpingography for detection of anatomical abnormalities of the genital tract. They also had blood drawn for testing for immunological risk factors, including antiphospholipid antibodies, antinuclear antibodies, antithyroid antibodies and lupus anticoagulant, to rule out other causes of recurrent miscarriage. Blood samples (5 ml) from the controls and RPL women were collected in ethylenediamine tetraacetic acid (EDTA)-coated collection vials and DNA was extracted using Qiagen DNA extraction kits (Qiagen, Hilden, Germany). This study was approved by the Eskişehir Ethics Committee No. 1. Written informed consent was obtained from all participants prior to registering for the study.

### Apo E genotyping

Fifty nanograms of genomic DNA was amplified in 25 liters PCR buffer containing 1 unit Taq DNA polymerase. A 228 bp fragment of the human Apo E gene was amplified with specific primers. The resulting PCR fragments were analyzed with a probe (ApoE C112R, detected in channel 530) and with probes labeled with LightCycler Red 640 (ApoE R158C, detected in channel 640). The Apo E codon 112 exhibits a Tm of 49.0°C in channel 530 for allele variant 112C and a Tm of 59.0°C in channel 530 for allele variant 112R. The Apo E codon 158 exhibits a Tm of 63.0°C in channel 640 for allele variant 158R and a Tm of 53.0°C in channel 640 for allele variant 158C. The use of a color compensation file generated with the LightMix kit - Color Compensation 530/640 is a prerequisite to run the duplex reaction. The supplied control DNA allows for the accurate comparison with unknown samples. aNot significant. Apo E, apolipoprotein E; RPL, recurrent pregnancy loss.

### Statistical analysis

The frequencies of gene mutations were compared between women experiencing RPL and the controls using a 2×2 contingency table with Fisher’s exact test (GraphPad InStat, San Diego, CA, USA). A two-tailed P-value <0.05 was considered to indicate a statistically significant difference.

## Results

Our study included 45 women with RPL and 45 women for the control group. The mean age of the RPL group was 33±0.78 years and the number of previous miscarriages ranged from 2 to 5. The mean age of the control group was 33.5±0.7 years and there was no significant difference between the groups (P>0.05). The mean number of weeks before pregnancy loss in the patient group was 8.3±0.32 weeks (range, 5–18). The mean birth time for the control group was 39.3±0.1 (range, 37–41) weeks.

The frequency of the Apo E genotypes for patients and controls are shown in Table I. In the RPL group, 66 chromosomes (73.3%) were positive for Apo E3, 16 (17.7%) were positive for Apo E4 and 8 (9%) were positive for Apo E2. In the control group, 60 chromosomes (66.6%) were positive for Apo E3, 18 (20%) were positive for Apo E4 and 12 (13.4%) were positive for Apo E2.

Apo E3 was the most frequent allele and Apo E2 was the rarest allele in the two groups. There was no significant difference in Apo E alleles in the two groups. Apo E2 was observed less in the patient group than in the control group; however, this was not statistically significant. There was no significant difference in Apo E genotype between the RPL and control groups. The most frequent genotype was Apo E3/E3 for the two groups.

**Figure 1:**
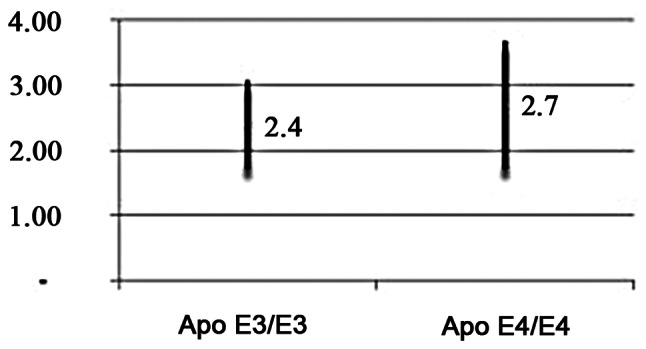
Mean rates of spontaneous abortion in the RPL group. RPL, recurrent pregnancy loss; Apo E, apolipoprotein E.

**Figure 2:**
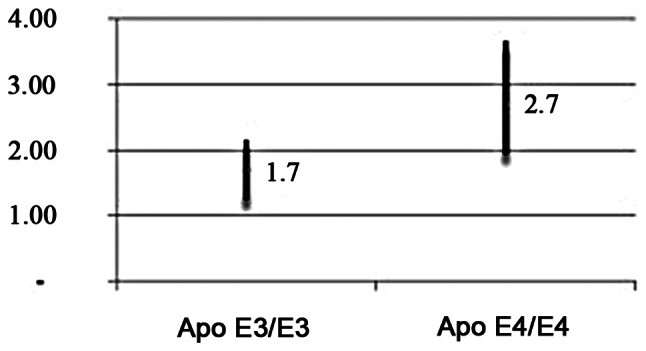
Mean pregnancy rates in the control group. Apo E, apolipoprotein E.

In the RPL group, the mean spontaneous abortion rate was 2.4±0.65 for the Apo E3/E3 genotype and 2.7±0.95 for the Apo E4/E4 genotype (P>0.05; Fig. 1). In the control group, the mean pregnancy rate was 1.7±0.43 for the Apo E3/E3 genotype and 2.8±0.83 for the Apo E4/E4 genotype. (P>0.05; Fig. 2).

## Discussion

In this study, we examined Apo E gene polymorphisms and their association with RPL. According to our results, there is no significant correlation between RPL and Apo E gene polymorphisms. The Apo E4 allele has been shown to be associated with an elevated risk of recurrent miscarriage in a previous study (6). Furthermore, Ergin *et al*(7) reported an increased prevalence of Apo E3/3 and E4/4 genotypes among Turkish women experiencing RPL. Conversely, a study conducted on 150 RPL patients and 160 controls reported that there was no association between Apo E polymorphisms and RPL (8). They concluded that the clinical management of RPL patients should not be affected by the presence or absence of Apo E polymorphisms.

Apo E genotype distribution differs among populations. Eichner *et al*(3) reviewed the frequencies of E2, E3 and E4 alleles in different populations and reported that these range from 0.02–0.13 for E2, 0.06–0.85 for E3 and 0.11–0.31 for E4. In our study, Apo E3 (73.3–66.6%) was the most frequent Apo E allele in the two groups. There were no significant differences in the frequency of Apo E alleles. The Apo E2 (9–13.4%) allele was the rarest gene in the two groups.

Apo E is a glycosylated protein characterized by its wide tissue distribution and multiple functions. Apo E polymorphisms have been extensively studied in a variety of diseases, including multiple sclerosis (9) and Alzheimer’s disease (10). Significantly higher plasma LDL concentrations have been observed in individuals with the Apo E4 allele. Elevated plasma lipid levels contribute to the pathogenesis of thrombus formation by accumulating in the intima of blood vessels whose endothelial cells have been activated by inflammatory cytokines (11). Apo E has also been shown to play an important role in the inflammatory response. Animals expressing the E4 allele have significantly greater systemic elevations of the pro-inflammatory cytokines, tumor necrosis factor (TNF)-α and interleukin (IL)-6 (12). As pregnancy is a hypercoagulable state, it is not surprising that the additive effect of an Apo E4 genotype superimposed on this hypercoagulable state increases the risk of clotting. Pregnant women have an increased risk of thromboembolism compared with non-pregnant women, with an underlying rate of venous thrombosis of ∼1/1,000 pregnancies (13).

The role of thrombophilia in the causation of RPL has been proven. Inherited thrombophilia is considered to be a multifactorial condition in which susceptibility is conferred by a number of genetic polymorphisms (14). This may explain why women who carry the thrombophilic mutation in one of the susceptibility genes do not develop the thrombotic complications. The impact of polymorphisms of the Apo E allele on reproductive outcome is being pursued and has yielded conflicting conclusions.

Corbo *et al*(15) identified that the lowest reproductive efficiency occurs with the presence of the E2 allele, intermediate with the E4 allele and highest with the E3 allele. Zetterberg *et al*(16) compared the frequency of Apo E alleles in spontaneous abortuses and the adult population and reported that the E4 allele may have embryo protective effects. In one of the studies, altered expression of the Apo E3 isoform has been implicated in women having pre-eclampsia during pregnancy, whereas another study observed no association between Apo E polymorphisms and a pre-eclampsia state (5,17). In another study, a mild to moderate association was observed in Apo E gene mutation in women with RPL compared to controls (18).

We conducted this study on a group of 45 RPL patients and their genotype frequencies were compared with those of 45 control samples. We observed no correlation between RPL and Apo E polymorphisms. Further studies should use an appropriate sample size. The confusion regarding the role of Apo E in RPL may be addressed through multicenter studies where samples of different ethnicities, using the same selection criteria for the RPL group and controls, are used.

In conclusion, our findings show that further studies on Apo E gene polymorphisms in RPL are not required. With growing awareness of the correlation between genetic factors influencing hemostasis and pregnancy-related disorders, documentation of thrombotic causes are important due to the potential of performing randomized controlled clinical trials to determine the effect of thromboprophylaxis in such cases.

## Figures and Tables

**Table I:** Distribution of Apo E alleles among RPL patients and controls.

Apo E allele	RPL, n (%)	Control, n (%)	P-value
2	8 (9)	12 (13.4)	0.342^a^
3	66 (73.3)	60 (66.6)	0.328^a^
4	16 (17.7)	18 (20)	0.703^a^

a Not significant. Apo E, apolipoprotein E; RPL, recurrent pregnancy loss.
